# Supplementary material for: Lack of harmonisation of greenhouse gases reporting standards and the methane emissions gap
Source: Nat Commun. 2025 Feb 11;16:1537. doi: 10.1038/s41467-025-56845-3 (PMC11814072; doi:10.1038/s41467-025-56845-3)
Supplement: Supplementary file 1 — Supplementary Information [file 41467_2025_56845_MOESM1_ESM.pdf]

Supplementary Information: Lack of harmonisation of greenhouse gases reporting standards and the methane emissions gap

Simone Cenci[a,3] and Enrico Biffis[b,4]

[a]*Institute for Sustainable Resources, University College London, London WC1H 0NN, UK*
[b]*Centre for Climate Finance and Investment and Department of Finance, Imperial College Business School, London SW7 2AZ, UK*

A Firm and country level data

To identify differences between firms that follow the GHG protocol guidelines (i.e., that use the most recent GWP_{100} published by the IPCC in their disclosure) and firms that do not, we collect data on firms' fundamentals from COMPUSTAT and Refinitiv. Specifically we define Size as the log of sales (SALE, in USD) adjusted for inflation (sourced from the Federal Reserve Bank of St Louis Database) ; Tangibility is property plant and equipment (PPENT, in USD) divided by book assets (AT, in USD), Profitability is Earnings Before Interests, Tax, Depreciation and Amortization (EBITDA in USD) over lagged book asset. Market leverage is long-term plus short-term debt (F.DebtTot) divided by market value of assets: total assets (F.TotAssets) – book equity (F.ShHoldEqCom) + market equity (F.MktCap), market to book is the market value of assets divided bay Total Book Asset. Finally, we collect information on the number of carbon management activities from the CDP Climate Change questionnaire. These include, for example, activities that aim to increase efficiency of production processes, fleets fuel efficiency, new product design, among others.

To merge CDP data to COMPUSTAT and Refinitiv we first merge on ISIN numbers. Then, we merge the left over companies using companies names. Firms that could not be matched by ISIN numbers and company names, were matched based on standardised company names obtained after removing punctuation, capitalisations, and common suffixes such as *corp*, *llc*, and *inc*. Out

[3]simone.cenci@ucl.ac.uk
[4]e.biffis@imperial.ac.uk

of the 2864 companies that report methane emissions and an IPCC GWP metric, we could merge 1642 with their financial information.

We also collect country-level policy data from the Policy Instruments for the Environment Database (PINE), a dataset from the OECD that track countries' environmental policies. Finally, we collect global energy price data from the World Bank.

B Probit model

We estimate the systematic differences between the characteristics of companies that follow the GHG protocol guidelines in their methane disclosure and those that do not with a Probit model where the dependent variable is zero if in year t firms c uses the most recent GWP_{100} in the disclosure, and one otherwise (the dependent variable measures the probability of deviation from the standard). All the firm-level independent variables, that include firms' Size, ratio of property plants and Equipment over total book assets (Tangibility), Profitability, Market-to-Book and Leverage are measured on a three years rolling basis up to the fiscal year in which the reported emission occurred. For robustness we also repeat the estimation with the fundamentals measured on a yearly basis and by running multiple regressions to estimate the effect of each set of variables independently.

In the regressions, we also control for current (Scope 1) emissions per unit of sales (i.e., Scope 1 emission from CDP over sales in USD), the number of environmental policies active in the country in the year of the emission, the country GDP and the World Bank Energy Price Index. The objective of introducing country-level controls (either through fixed effects or policy variables) is to control for systematic deviation from IPCC GWPs due to factors exogeneous to the firms.

Because disclosure to CDP is a voluntary process our sample is subject to self-selection bias. To correct for this bias we estimate the model with the Heckman correction[38]. Specifically, we run a

two-stage model. The first model is a Probit where the dependent variable is a binary indicator that takes the value of one if company c discloses emission to the CDP questionnaire in year t and zero otherwise. The independent variables include Profitability, Size, Tangibility, year-fixed effects. We also control for the proportion of companies in any single country and sector that disclose emission to CDP. These two factors are necessary for correct identification using the Heckman correction because they influence the selection equation but not the outcome[39]. We call the control set of the Probit model $\tilde{\mathbf{X}}$ to distinguish it from the control set of the main regressions. From the fitted Probit model, we estimate the inverse Mills ratio, $\mathcal{M}$, which is defined as: $\mathcal{M} = \frac{f(x)}{F(x)}$ where $f(x), F(x)$ are the (normal) probability density function and the cumulative distribution, respectively. Then we use the inverse Mills ratio from the Probit as an additional covariate in the main Probit. Notice that whilst the Heckman correction was originally derived using an OLS estimator in the second stage model, the statistical properties of the correction are preserved under a Probit[40].

$$\mathbb{P}[\text{Disclosure}_t|\tilde{\mathbf{X}}_t] = \Phi(\tilde{\mathbf{X}}_t^T\tilde{\boldsymbol{\beta}}) \tag{6}$$

$$\text{A)}\ \mathbb{P}[\text{Deviation from latest GWP}_{100,t}|\{\mathbf{X}_{c,\langle t_3\rangle}, \mathcal{L}_{g,t}, \mathcal{P}_t, \mathcal{S}, \mathcal{M}_{c,t}, \mathcal{D}_t, \mathcal{A}_{c,t}\}] = \Phi(c+ \tag{7}$$
$$+\,\alpha\mathbf{X}_{c,\langle t_3\rangle} + \beta\mathcal{L}_{g,t} + \gamma\mathcal{P}_t + \delta\mathcal{M}_{c,t} + \omega\mathcal{S} + \eta\mathcal{D}_t + \zeta\mathcal{A}_{c,t})$$

$$\text{B)}\ \mathbb{P}[\text{Deviation from latest GWP}_{100,t}|\{\mathbf{X}_{c,\langle t_3\rangle}, \mathcal{S}, \mathcal{R}, \mathcal{Y}, \mathcal{M}_{c,t}, \mathcal{D}_t, \mathcal{A}_{c,t}\}] = \Phi(c + \alpha\mathbf{X}_{c,\langle t_3\rangle}+ \tag{8}$$
$$+\,\beta\mathcal{R} + \gamma\mathcal{Y} + \delta\mathcal{M}_{c,t} + \omega\mathcal{S} + \eta\mathcal{D}_t + \zeta\mathcal{A}_{c,t})$$

$$\text{C)}\ \mathbb{P}[\text{Deviation from latest GWP}_{100,t}|\{\mathbf{X}_{c,t}, \mathcal{R}, \mathcal{C}, \mathcal{Y}, \mathcal{M}_{c,t}, \mathcal{D}_t, \mathcal{A}_{c,t}\}] = \Phi(c + \alpha\mathbf{X}_{c,t} + \beta\mathcal{R}+ \tag{9}$$
$$+\,\gamma\mathcal{Y} + \delta\mathcal{M}_{c,t} + \omega\mathcal{S} + \eta\mathcal{D}_t + \zeta\mathcal{A}_{c,t})$$

Where $\mathbf{X}_{c\langle t_3\rangle}$ are company-level data measured as rolling averages over the previous three fiscal years (the subscript c denotes company level variables); $\mathcal{L}_{g,t}$ are country-level data (i.e., environmental policies, GDP); $\mathcal{P}_t$ is the Energy Price Index, a global-level variable; $\mathcal{S}$ are sector fixed effects; $\mathcal{M}_{c,t}$ is the inverse Mills Ratio estimated from the Probit in Eq. (6); $\mathcal{D}_t$ is the distance, in years, from

the release of the latest GWP_{100} ; $\mathcal{A}_{c,t}$ is the number of carbon management activities implemented and reported in year t. We then repeat the estimation using regional and year fixed-effect $(\mathcal{R}, \mathcal{Y})$ as opposed to country and global level data (Eq. (8)).

As a further robustness test, in the last regression (Eq. (9)) we measure financial fundamentals in the same year of the disclosure instead of including them in the model as historical averages. When using yearly data, there is a clear directionality in the relationship between the control variables. For example, in any given year revenue drives earnings which are used to measure Profitability. Hence including them both in the regression leads to unfair comparison of coefficients as one variable blocks the path of the other[41–43]. To fairly compare the coefficients we need to estimate multiple regression specifications each for each variable that plays a comparable role in the underlying data generating process. Specifically, we first estimate the effect of Size, controlling for self-selectivity, previous year book asset, year,country and sector fixed effects, as none of the other variables drive revenue on a contemporaneous basis. Then we add Profitability, Tangibility, and Market-to-Book (which are driven by, but do not drive, revenue and book value of assets) to the model, and finally we add Leverage, Current Emissions and the number of carbon management activities reported to CDP.

The results of the three regressions are shown in table ST1. The table shows that companies that are more likely to deviate from the GWP_{100} standard are larger, have predominantly tangible assets and have a low predicted growth (i.e., low "Market-to-Book" ratio). Interestingly, deviations are also more likely for companies that implement a lower number of carbon management activities as reported to the CDP climate change questionnaire, and, as expected, become less likely as new standards become established ("Time from new standard", i.e., the distance, in years, from the release of the latest GWP_{100}).

[38] Heckman, J. J. Sample selection bias as a specification error. *Econometrica* **47**, 153–161 (1979).

	Main regression	With fixed-effects	Yearly measurements
Size	0.06***	0.05***	0.04***
Profitability	-0.0	-0.0	-0.0
Tangibility	0.03***	0.03***	0.02***
Market-to-Book	-0.03***	-0.03***	-0.02**
Leverage	-0.01	-0.02*	-0.02**
# Env. Policies	-0.01		
Current emissions	-0.01*	-0.01*	-0.01
GDP	0.02**		
Time from new standard	-0.08***		
Energy Price Index	0.14***		
Carbon management activities	-0.01**	-0.01**	-0.01**

Table ST1: | **Systematic differences between companies that follow standard carbon accounting guidelines and companies that do not.** The table shows the marginal effects from the Probit model for the main regression (Eq. (7), N=4518), the fixed-effects regression (Eq. (8), N=4518), and the regression that uses yearly measures of fundamentals (as opposed to their three-year averages, Eq. (9), N=4593). The dependent variable is zero if in year t firms c adopts the most recent GWP_{100}, and one otherwise. Hence, positive coefficients denote higher probabilities of deviating from the GHG protocol guidelines. *,**,*** denote statistical significance at 10%,5%, and 1%, respectively.

[39] Wolfolds, S. E. & Siegel, J. Misaccounting for endogeneity: The peril of relying on the heckman two-step method without a valid instrument. *Strategic Management Journal* **40**, 432–462 (2019).

[40] Van de Ven, W. P. M. M. & Van Praag, B. M. S. The demand for deductibles in private health insurance: A probit model with sample selection. *Journal of Econometrics* **17**, 229–252 (1981).

[41] Keele, L., Stevenson, R. T. & Elwert, F. The causal interpretation of estimated associations in regression models. *Political Science Research and Methods* **8**, 1–13 (2020).

[42] Hünermund, P. & Louw, B. On the nuisance of control variables in causal regression analysis. *Organizational Research Methods* 10944281231219274 (2023).

[43] Cenci, S. Overlooked biases from misidentifications of causal structures. *The Journal of Finance and Data Science* **10**, 100127 (2024).

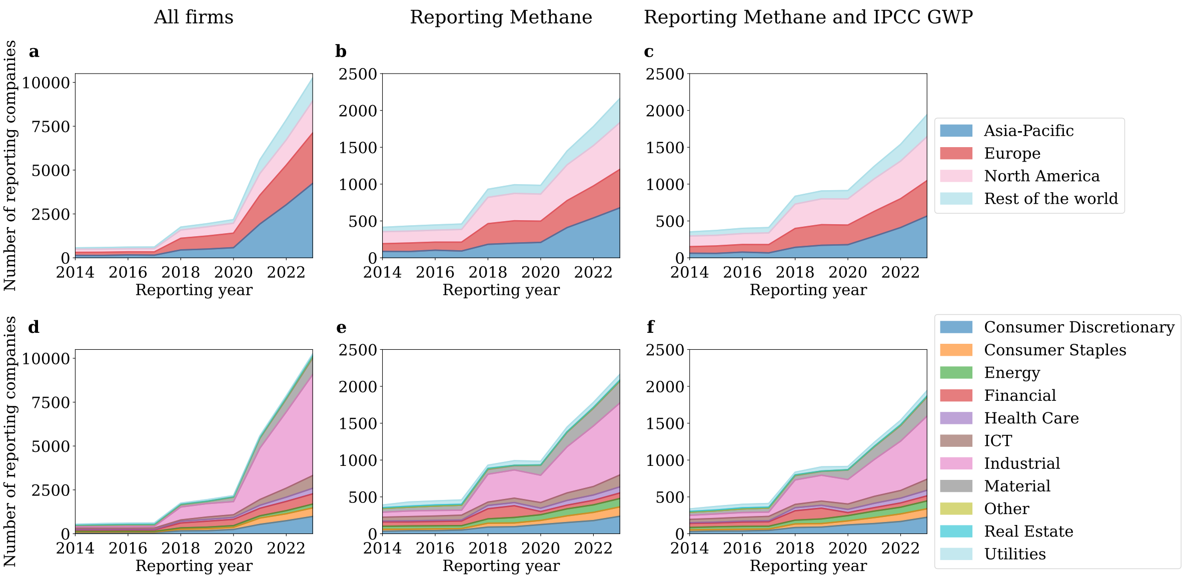

Fig. S1 | Number of companies in the sample by year, geography, and sector. The figure shows the number of firms by geography (panel **a,b,c**) and sector (panel **d,e,f**) that reports to CDP (panel **a,d**), that also reports methane emissions (panel **b,e**) and that also explicitly reports an IPCC GWP value (panel **c,f**). Details on sectors and geography can be found in tables ST4 and ST5, respectively.

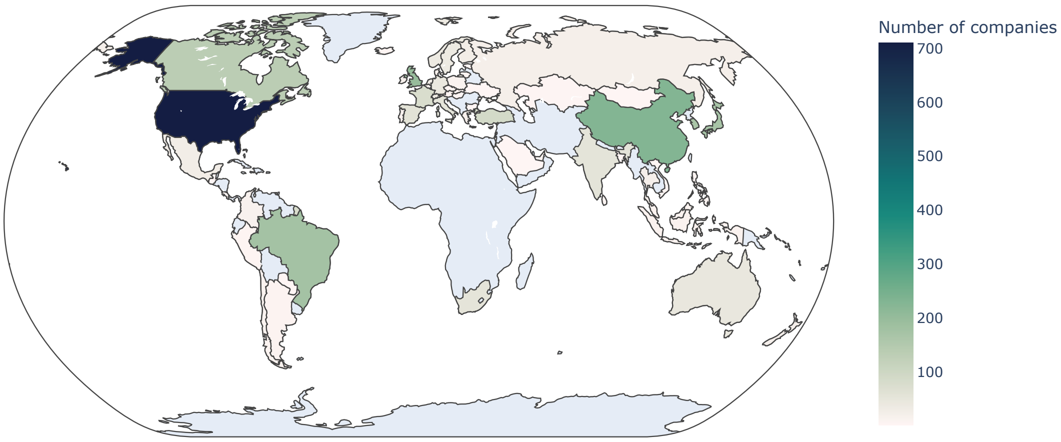

Fig. S2 | Geographical distribution of companies in the sample. The figure shows the distribution of the number of companies in the subsample with available data on methane emissions and GWP factors. Gray countries are missing from the sample.

Report	20-year	100-year
SAR	56.0	21.0
TAR	62.0	23.0
AR4	72.0	25.0
AR5	84.0	28.0
AR6	[79.7, 81.1, 82.5]	[27., 28.4, 29.8]

Table ST2: | **Global Warming Potential values.** The table shows the GWP values used in the main text to convert CH_4 emissions in CO_2e units. SAR, AR4 and AR5 are sourced from the GHG protocol at https://ghgprotocol.org/sites/default/files/ghgp/Global-Warming-Potential-Values%20%28Feb%2016%202016%29_1.pdf. TAR is sourced from Table 1 in https://unfccc.int/resource/docs/tp/tp0403.pdf. The left and right extremes in AR6 denote non-fossil and fossil methane, respectively, as from table 7.15 in (IPCC, 2021: Climate Change 2021: The Physical Science Basis. Contribution of Working Group I to the Sixth Assessment Report of the Intergovernmental Panel on Climate Change [Masson-Delmotte, V., P. Zhai, A. Pirani, S.L. Connors, C. Péan, S. Berger, N. Caud, Y. Chen, L. Goldfarb, M.I. Gomis, M. Huang, K. Leitzell, E. Lonnoy, J.B.R. Matthews, T.K. Maycock, T. Waterfield, O. Yelekçi, R. Yu, and B. Zhou (eds.)]. Cambridge University Press, Cambridge, United Kingdom and New York, NY, USA, 2391 pp.).The central value in the AR6 GWP are the mean values used for the analyses in the main text.

GWP source	Reporting ear
Other: The Climate Registry: General Reporting Protocol Version 2.0	2014
Other: All emission factors sourced from EPA's Emission Factors Hub, April 2014 (http://www.epa.gov/climateleadership/inventory/ghg-emissions.html)	2015
Other, please specify: Defra, Fuels Conversion Factors 2014	2020
Other: GHG indicator	2015
Other, please specify: GHG Protocol (Emissions factors from cross-sector tools) [March 2017]	2023
Other, please specify: Calculated CO2 emissions using MMBtu x fuel specific CO2 emission factors from 40 CFR Part 98 Subpart C Table C-1 (revised Nov 2013)	2022
Other, please specify: e-CFR as of August 15 2018	2023
Other, please specify: As per National Government regulation	2023
Other: EPA Mandatory Reporting of Greenhouse Gases-Final Rule, 40 CFR Part 98, Table A-1	2014
Other, please specify: DEFRA_Conversion factors_2020	2021
Other: Base Carbone ADEME, January 2014	2014
Other, please specify: WRI, WBCSD, NCASI wood Products worksheet	2019
Other, please specify: Scope 1: Natural gas shrinkage (Leakage +Theft of Gas+ Own use of gas) reported as tCO2e, but since natural gas is predominantly methane, any emissions are equivalent. Reporting in accordance with Ofgem Guidance: OFGEM GD-2RIGs.	2023
Other: GHG Protocol Tool 2011	2015
Other, please specify: Other, please specify:	2019

Table ST3: **Examples of GWP sources excluded from the analysis** The table provides a few examples of GWP sources that we have excluded from the analysis because we could not systematically map them into IPCC values.

hotels restaurants and leisure and tourism services	Consumer Discretionary
automobiles and components	Consumer Discretionary
consumer durables household and personal products	Consumer Discretionary
homebuilding	Consumer Discretionary
hospitality	Consumer Discretionary
apparel	Consumer Discretionary
retail	Consumer Discretionary
Education services	Consumer Discretionary
Dealers, wholesalers & distributors	Consumer Discretionary
Travel services	Consumer Discretionary
Consumer goods wholesale & rental	Consumer Discretionary
retailing	Consumer Discretionary
Animal products wholesale	Consumer Discretionary
specialized consumer services	Consumer Discretionary
textiles apparel footwear and luxury goods	Consumer Discretionary
tires	Consumer Discretionary
Consumer services	Consumer Discretionary
food and beverage processing	Consumer Staples
food and staples retailing	Consumer Staples
food beverage and agriculture	Consumer Staples
Agricultural products wholesale	Consumer Staples
Food & beverage wholesale	Consumer Staples
fossil fuels	Energy
oil and gas	Energy
Energy services & equipment	Energy
Insurance	Financial
Other financial	Financial
REIT	Financial
Asset managers	Financial
banks diverse financials insurance	Financial
banks diverse financials and insurance	Financial
Banks	Financial
healthcare providers and services and healthcare technology	Health Care
health care providers and services and healthcare technology	Health Care
pharmaceuticals biotechnology and life sciences	Health Care
healthcare equipment and supplies	Health Care
biotech health care and pharma	Health Care
Pharma & health care supplies wholesale & distribution	Health Care
Health care services	Health Care
software and services	ICT
IT services	ICT
media	ICT
technology hardware and equipment	ICT
Media	ICT
telecommunication services	ICT
Print publishing	ICT
Servers & data centers	ICT
semiconductors and semiconductors equipment	ICT
Web-based services	ICT
Telecommunications services	ICT

Software	ICT
Technology hardware wholesale & distribution	ICT
construction and engineering	Industrial
Engineering services	Industrial
professional services	Industrial
Commercial services	Industrial
aerospace and defense	Industrial
Transportation equipment wholesale & dealing	Industrial
manufacturing	Industrial
infrastructure	Industrial
Industrial machinery distribution	Industrial
Industrial services	Industrial
Transportation support services	Industrial
Other professional services	Industrial
building products	Industrial
trading companies and distributors and commercial services and supplies	Industrial
electrical equipment and machinery	Industrial
Printing services	Industrial
Vehicles & machinery rental & leasing	Industrial
transportation	Industrial
Chemicals wholesale & distribution	Material
materials	Material
forest and paper products - forestry timber pulp and paper rubber	Material
Mining & metals support services	Material
mining	Material
Biofuel supply	Material
Wood & paper products wholesale	Material
Construction & building materials dealing & distribution	Material
containers and packaging	Material
chemicals	Material
construction materials	Material
mineral extraction	Material
Marketing	Other
international bodies	Other
corporate tags	Other
real estate	Real Estate
Real estate services	Real Estate
services	Services
power generation	Utilities
gas utilities	Utilities
electric utilities and independent power producers and energy traders (including fossil alternative and nuclear energy)	Utilities
water utilities	Utilities

Table ST4: **Sector mapping** The table shows the mapping from sector and primary activities reported to the CDP Climate Change Questionnaire and the Global Industry Classification Standard

Country_adj	Geography
Afghanistan	Asia-Pacific
Hong Kong	Asia-Pacific
Japan	Asia-Pacific
Viet Nam	Asia-Pacific
Korea (the Republic of)	Asia-Pacific
Malaysia	Asia-Pacific
Fiji	Asia-Pacific
Marshall Islands (the)	Asia-Pacific
Mongolia	Asia-Pacific
New Zealand	Asia-Pacific
Norfolk Island	Asia-Pacific
Indonesia	Asia-Pacific
Philippines (the)	Asia-Pacific
China	Asia-Pacific
Cambodia	Asia-Pacific
Pakistan	Asia-Pacific
India	Asia-Pacific
Bangladesh	Asia-Pacific
Sri Lanka	Asia-Pacific
Australia	Asia-Pacific
Tuvalu	Asia-Pacific
Singapore	Asia-Pacific
Thailand	Asia-Pacific
Italy	Europe
Ukraine	Europe
Jersey	Europe
Luxembourg	Europe
Isle of Man	Europe
United Kingdom of Great Britain and Northern Ireland (the)	Europe
Latvia	Europe
Ireland	Europe
Liechtenstein	Europe
Lithuania	Europe
San Marino	Europe
Serbia	Europe
Sweden	Europe
Monaco	Europe
Netherlands (the)	Europe
Spain	Europe
Norway	Europe
Slovenia	Europe
Slovakia	Europe
Poland	Europe
Portugal	Europe
Romania	Europe
Russian Federation (the)	Europe
Switzerland	Europe
Malta	Europe
Åland Islands	Europe

Finland	Europe
Hungary	Europe
Belgium	Europe
Belarus	Europe
Estonia	Europe
Croatia	Europe
Guernsey	Europe
Iceland	Europe
Bulgaria	Europe
Czechia	Europe
France	Europe
Greece	Europe
Germany	Europe
Denmark	Europe
Austria	Europe
Canada	North America
Greenland	North America
United States of America (the)	North America
Bermuda	North America
Bolivia (Plurinational State of)	Rest of the world
Saudi Arabia	Rest of the world
Cayman Islands (the)	Rest of the world
Brazil	Rest of the world
Qatar	Rest of the world
Puerto Rico	Rest of the world
Cameroon	Rest of the world
Iraq	Rest of the world
Chile	Rest of the world
Bahrain	Rest of the world
Azerbaijan	Rest of the world
Trinidad and Tobago	Rest of the world
Tunisia	Rest of the world
Turkey	Rest of the world
Argentina	Rest of the world
Angola	Rest of the world
United Arab Emirates (the)	Rest of the world
Algeria	Rest of the world
Uruguay	Rest of the world
Venezuela (Bolivarian Republic of)	Rest of the world
South Africa	Rest of the world
Colombia	Rest of the world
Oman	Rest of the world
Peru	Rest of the world
Honduras	Rest of the world
Israel	Rest of the world
Guyana	Rest of the world
Jamaica	Rest of the world
Guatemala	Rest of the world
Jordan	Rest of the world
Kazakhstan	Rest of the world
Kuwait	Rest of the world
Ghana	Rest of the world
Lebanon	Rest of the world

Libya	Rest of the world
Georgia	Rest of the world
Costa Rica	Rest of the world
Madagascar	Rest of the world
Mauritania	Rest of the world
Mauritius	Rest of the world
Mexico	Rest of the world
El Salvador	Rest of the world
Egypt	Rest of the world
Morocco	Rest of the world
Mozambique	Rest of the world
Ecuador	Rest of the world
Nigeria	Rest of the world
Cyprus	Rest of the world
Panama	Rest of the world
Paraguay	Rest of the world
Equatorial Guinea	Rest of the world
Kenya	Rest of the world

Table ST5: **Geographical mapping** The table shows the mapping from country to macro geographical regions

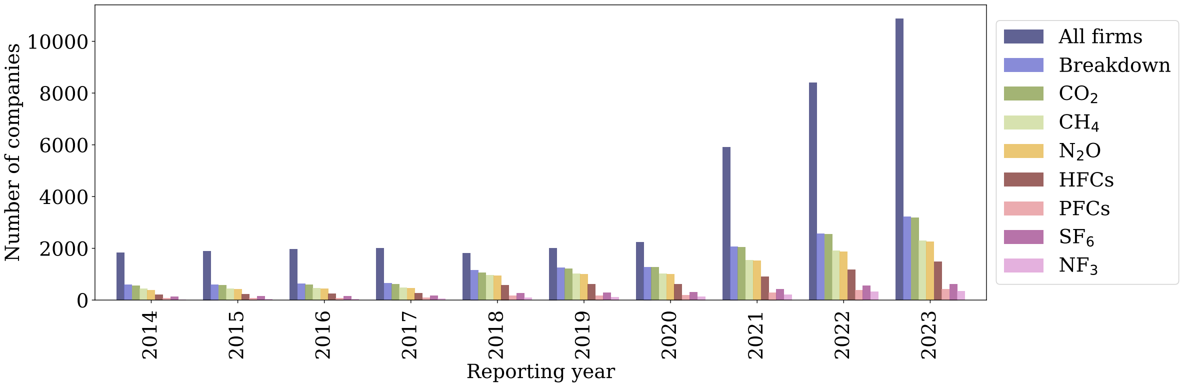

Fig. S3 | Breakdown of the non-CO_2 disclosure The figure shows the number of companies in the CDP dataset alongside the number of companies that breakdown Scope 1 emissions into individual GHG and the count of companies disclosing information in each gas.

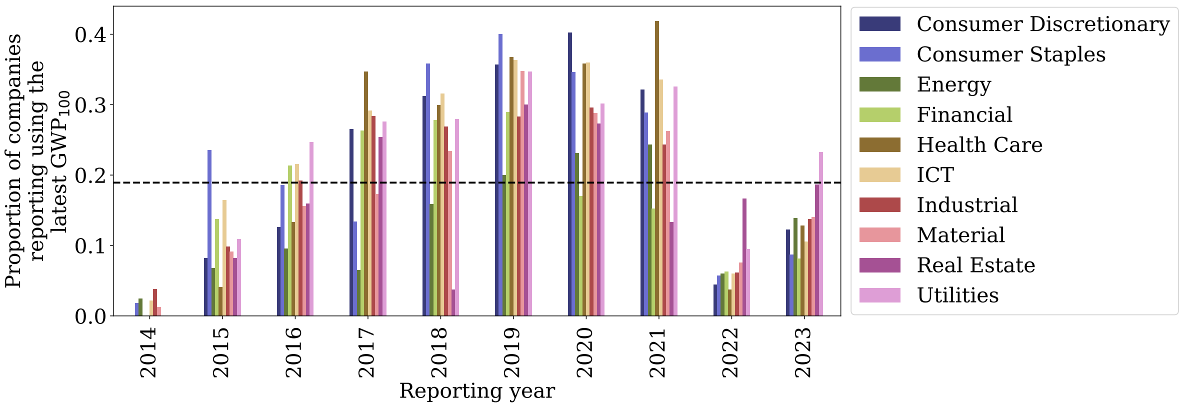

Fig. S4 | Proportion of companies reporting using the latest GWP_{100} The figure shows the proportion of companies, by sector, that report non-CO_2 emissions using the latest GWP_{100} as recommended by most reporting guidelines. The dotted horizontal line shows the sample mean.

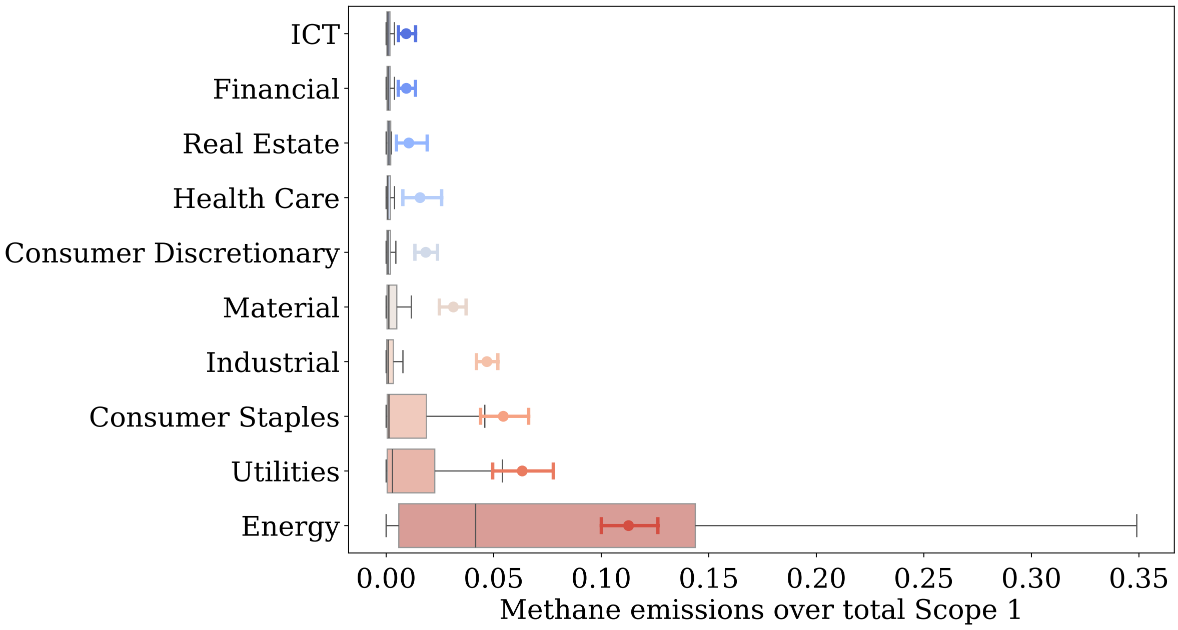

Fig. S5 | Methane intensity by sector. The figure shows the distribution of the ratio of methane emissions over total Scope 1 emissions by sector. Scope 1 emissions are those reported by the companies to CDP before breakdown into individual GHGs. As expected, the Energy and Consumer Staples (which includes Agriculture, see ST4) sectors are the most methane intensive sectors. The lines within the box plots are median lines, the full circles are the means of the distributions, error bars are their standard errors, and the edges of the boxes are the quartile range: the 25th and 75th percentile (N = 9703).

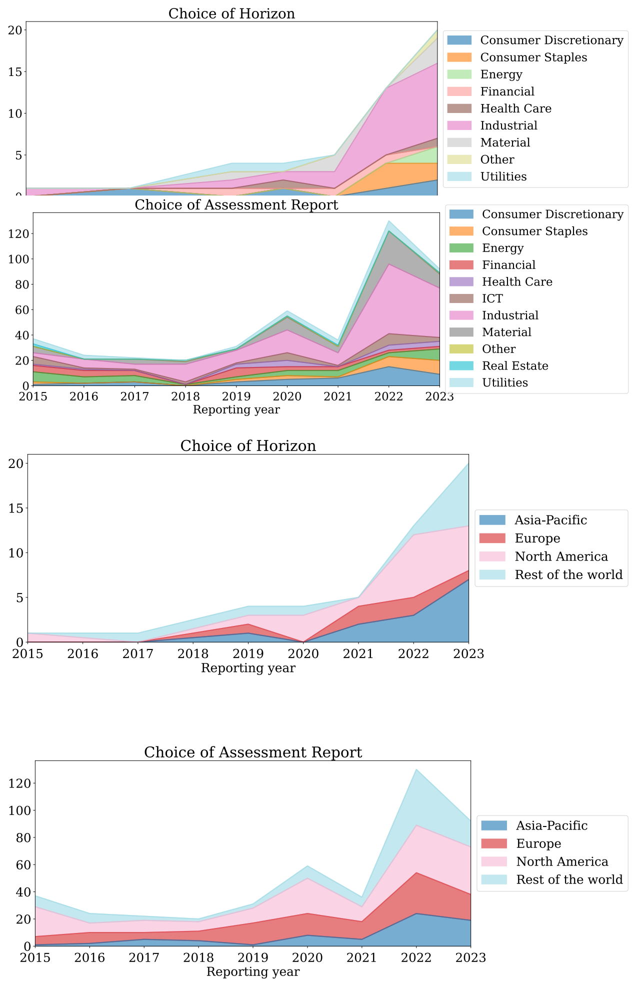

Fig. S6 | Sectoral and geographical decomposition of choice behaviour under the GWP$_{100}$ counterfactual The figure shows the sectorial and geographical decomposition of the number of companies that change emission metric or AR source in any given year. Overall, we do not observe a substantial sector or geography bias in the sample.

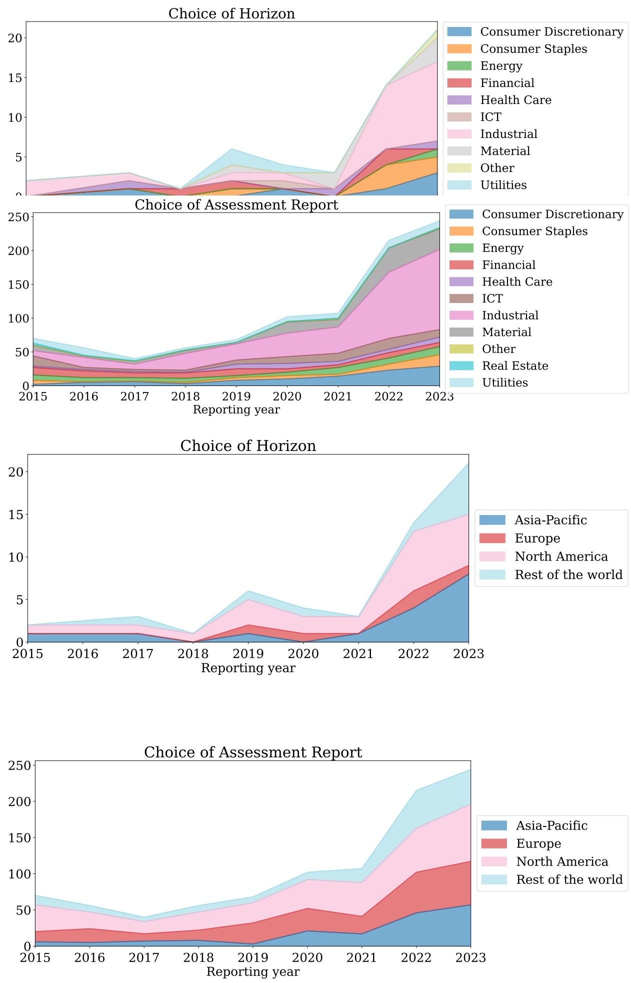

Fig. S7 | Sectoral and geographical decomposition of choice behaviour under the GWP_{20} counterfactual The figure shows the sectorial and geographical decomposition of the number of companies that change emission metric or AR source in any given year. The numbers are different from those reported in figure S6 because far fewer companies in the sample switch to the most recent GWP_{20} . Overall, we do not observe a substantial sector or geography bias in the sample.

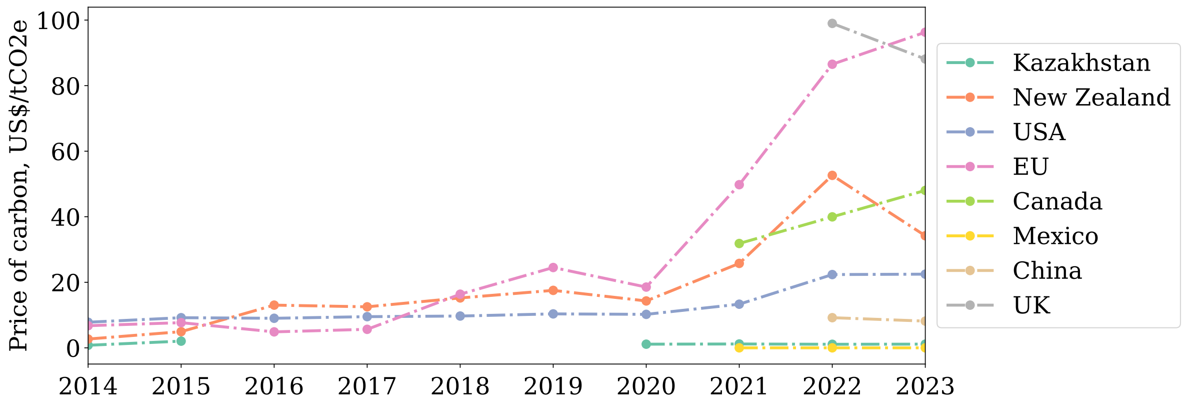

Fig. S8 | Carbon price. The figure shows the price of carbon used in the economic cost analyses in the main text. Data are from the world bank available at https://carbonpricingdashboard.worldbank.org/. For countries with both national and regional ETSs we use the price of carbon from the national market. For countries with only regional (or state) ETSs, such as the US, for example, where only a few states have active carbon pricing mechanisms, we use the average carbon price as national carbon price. For countries in the European union we use carbon prices from the EU ETS.

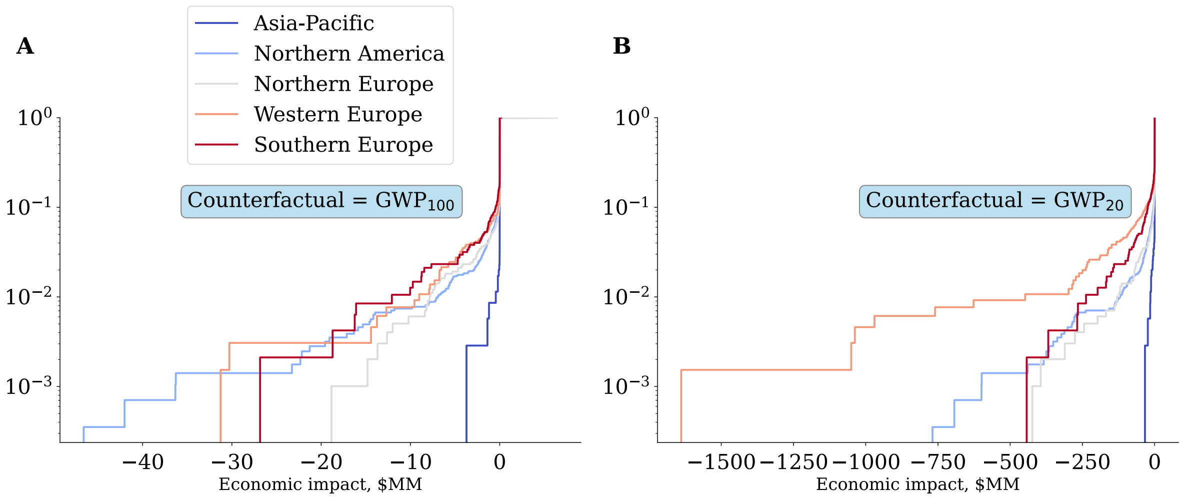

Fig. S9 | Geographical distribution of economic impacts. The figure show the distribution of economic impact by macro region. Note that the we have grouped together companies in Central and Eastern Asia with companies in Australia and New Zealand to balance the size of each group.

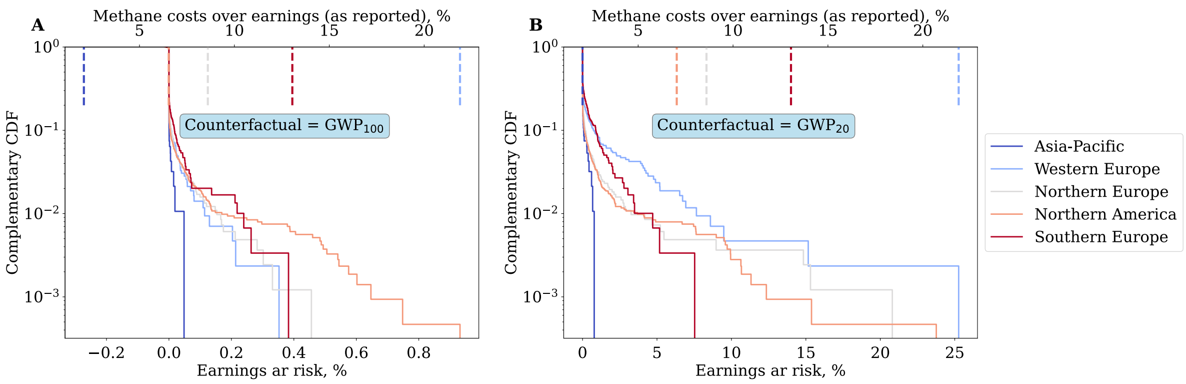

Fig. S10 | Geographical distribution of transition risks. The figure show the distribution of transition risks by macro region. Note that the we have grouped together companies in Central and Eastern Asia with companies in Australia and New Zealand to balance the size of each group.
